# Super-Resolution Contrast-Enhanced Ultrasound Methodology for the Identification of In Vivo Vascular Dynamics in 2D

**DOI:** 10.1097/RLI.0000000000000565

**Published:** 2019-05-02

**Authors:** Evangelos Kanoulas, Mairead Butler, Caitlin Rowley, Vasiliki Voulgaridou, Konstantinos Diamantis, William Colin Duncan, Alan McNeilly, Michalakis Averkiou, Hessel Wijkstra, Massimo Mischi, Rhodri Simon Wilson, Weiping Lu, Vassilis Sboros

**Affiliations:** From the * Institute of Biochemistry, Biological Physics, and Bio Engineering, and; † Department of Physics, Heriot-Watt University, Riccarton;; ‡ Institute for Digital Communications, and; § Center for Reproductive Health, University of Edinburgh, Edinburgh, United Kingdom,; ∥ Department of Bioengineering, University of Washington, Seattle, WA;; ¶ Department of Urology, AMC, University Hospital, Amsterdam;; # Department of Electrical Engineering, Eindhoven University of Technology, Eindhoven, the Netherlands; and; 8 **Oxford Institute for Radiation Oncology, Department of Oncology, University of Oxford, Oxford, United Kingdom.

**Keywords:** microbubbles, detection, localization, tracking, ultrasound, biomedical imaging, super-resolution, prostate cancer

## Abstract

**Objectives:**

The aim of this study was to provide an ultrasound-based super-resolution methodology that can be implemented using clinical 2-dimensional ultrasound equipment and standard contrast-enhanced ultrasound modes. In addition, the aim is to achieve this for true-to-life patient imaging conditions, including realistic examination times of a few minutes and adequate image penetration depths that can be used to scan entire organs without sacrificing current super-resolution ultrasound imaging performance.

**Methods:**

Standard contrast-enhanced ultrasound was used along with bolus or infusion injections of SonoVue (Bracco, Geneva, Switzerland) microbubble (MB) suspensions. An image analysis methodology, translated from light microscopy algorithms, was developed for use with ultrasound contrast imaging video data. New features that are tailored for ultrasound contrast image data were developed for MB detection and segmentation, so that the algorithm can deal with single and overlapping MBs. The method was tested initially on synthetic data, then with a simple microvessel phantom, and then with in vivo ultrasound contrast video loops from sheep ovaries. Tracks detailing the vascular structure and corresponding velocity map of the sheep ovary were reconstructed. Images acquired from light microscopy, optical projection tomography, and optical coherence tomography were compared with the vasculature network that was revealed in the ultrasound contrast data. The final method was applied to clinical prostate data as a proof of principle.

**Results:**

Features of the ovary identified in optical modalities mentioned previously were also identified in the ultrasound super-resolution density maps. Follicular areas, follicle wall, vessel diameter, and tissue dimensions were very similar. An approximately 8.5-fold resolution gain was demonstrated in vessel width, as vessels of width down to 60 μm were detected and verified (λ = 514 μm). Best agreement was found between ultrasound measurements and optical coherence tomography with 10% difference in the measured vessel widths, whereas ex vivo microscopy measurements were significantly lower by 43% on average. The results were mostly achieved using video loops of under 2-minute duration that included respiratory motion. A feasibility study on a human prostate showed good agreement between density and velocity ultrasound maps with the histological evaluation of the location of a tumor.

**Conclusions:**

The feasibility of a 2-dimensional contrast-enhanced ultrasound-based super-resolution method was demonstrated using in vitro, synthetic and in vivo animal data. The method reduces the examination times to a few minutes using state-of-the-art ultrasound equipment and can provide super-resolution maps for an entire prostate with similar resolution to that achieved in other studies.

Ultrasound imaging is an indispensable tool in medical diagnosis,^1^ primarily due to its cost-effectiveness and unique real-time capability. Contrast-enhanced ultrasound (CEUS) imaging aims to assess vascular flow and tissue perfusion. This requires the intravenous injection of gas-filled microbubbles (MBs), which are efficient point scatterers of ultrasound.^2,3^ Their diameter, typically around 2 to 3 μm, allows passage through the entire vascular bed. Their nonlinear response to ultrasound has been deployed with specially designed ultrasound transmit pulse sequences to enhance their signals while also suppressing tissue signals.^1^ However, after more than 20 years of research, there are very few CEUS applications in the clinic that are supported by health services around the world. The most important application is in the differential diagnosis of liver lesions,^4–7^ whereas other applications in the abdomen,^8–10^ cardiovascular,^11–14^ or using targeted and therapeutic MBs^15–19^ are rather research oriented or have limited clinical use. The main reason for the limited use of CEUS is the high interobserver and intraobserver variability compared with that what is achieved by other imaging modalities (eg, magnetic resonance imaging [MRI] or computed tomography [CT]). Although ultrasound imaging provides comparable resolution to MRI or CT, typically 0.3 to 1 mm for abdominal applications, a number of factors including the equipment type and settings, the patient variability, and contrast material^20^ affect its performance. All of the previously mentioned items have a significant role in the increased uncertainty of CEUS-derived measurements of blood volume and flow that are rather not quantitative.^21,22^ Despite their limited temporal resolution, MRI and CT are often preferred.

A recent advance, with significant potential in medical imaging, is the application of particle localization and tracking methodologies to CEUS, often inspired by light microscopy.^23–26^ Despite their small size compared with the ultrasound wavelength (typically around 100 times smaller), most MBs have high scattering cross-section (SCS) and provide strong ultrasound scatter that can be recorded as a single point spread function (PSF) in the image.^27–29^ Thus, their localization is possible, and early in vivo images showed at least a 5- and up to 10-fold resolution improvement at diagnostic frequencies (6.5 MHz).^24,30,31^ The work used a small number of single MBs that can be detected and then tracked. Algorithms for particle detection and localization can be implemented in ultrasound data.^23,24,30,32–34^ The focus is on localization of single MBs either isolated^30,35,36^ or with enough separation between echoes within a group of multiple MBs, and often overlapping MB echoes, due to MBs located closely, are rejected.^24^

In practice, the image plane includes a large dispersion of vessel diameters that range from a few micrometers and up to millimeters. The MB concentration will be linearly related to the volume of these different-sized vessel groups, and consequently, under conditions of constant volume flow, the number of MBs that cross any given microvessel will be a few orders of magnitude lower than the feeder vessel, which is millimeters in diameter. An infusion of contrast agent that ensures a small number of MBs in any given frame is likely to ensure single MB scattering events that are easy to detect, localize, and track. However, given the large number of microvessels and the need for enough MBs to cross the entire vasculature available in the image, it would be necessary to acquire long duration video loops in the order of tens of minutes or possibly hours depending on the application, size of organ, or region of interest. Such data sets render examination procedures impractical and are likely to provide significant motion artifacts that may be several magnitudes of orders larger than the aimed resolution. To alleviate this problem, the length of video data is required to reduce to a few minutes and thus the concentration of MBs must be increased. Tracking of a large number of MBs is also possible.^24,25,31,32,35^ The most efficient method is provided by high frame rate imaging that can provide scatter overlap for flowing MBs and deploy tracking using a simple image analysis method that follows the MB path by means of autocorrelation.^23,26^ This means that all single MBs will be tracked and the tracking efficiency is near 100%. To achieve high frame rate, single-emission protocols, for instance plane waves, were used. This provides a high acoustic pressure that drops significantly with depth and normally a high acoustic pressure near the transducer, which has 2 consequences: (1) the penetration depth is limited as there is no focus to offset the attenuation, and (2) the MB detection sensitivity is highly variable. This is due to the highly variable acoustic pressure with depth and the dependence of the MB SCS on acoustic pressure^27,37^ (ie, SCS increases with increasing acoustic pressure). In general, higher acoustic pressures provide larger MB densities as a larger number of MBs scatter above noise. In addition, if the acoustic pressure is high then there will be significant MB destruction. Thus, the assumption that the MB density across the image is uniform and representative of the vascular volume is difficult to uphold.

A near-uniform pressure field can be achieved by using a focused transmission. It is possible to approximately offset the attenuation using a focus at the bottom of the image. Although the attenuation is variable at different parts of the image, this approximation ensures a near-uniform distribution of MBs across the image that provides a good approximation of the concentration of red blood cells and hence blood volume. Currently, ultrasound imaging systems use nearly exclusive focused transmissions, and it is also possible to generate ultrasound images with single MBs using these systems. However, the frame rate will be low in the order of 10 to 20 Hz. This makes MB tracking challenging as MB echoes across subsequent frames usually show no overlap, complicating the reconstruction of the MB trajectories. Also note that the speed of red blood cells ranges from micrometers to centimeters per second.^38,39^ Thus, in the case of large MB density in the image, it is difficult to identify each MB in consecutive frames. Furthermore, a large MB density will provide several MB events that are merged and not separated. Although the focus on isolating single MBs^24^ ensures good localization results and thus optimal resolution outcome, the merged MB events are data that should be used to maximize data usage and reduce video loop duration. More recently, sparse recovery methods seem promising in providing similar quality information by using large MB densities and deploying prior knowledge of the PSF.^40,41^ Here we use lower MB densities that would enable their separation but would also create several merged MB scatter events in the image. It is assumed that these merged MB events are likely to be in image regions of larger vessels where the flow is pulsatile, hence the MB density is high. On the other hand, microvessels may provide mostly single MB events as the blood volume drops and the velocity profiles are nearly close to an average.

A model-based approach for tracking a large number of particles has been developed for tracking single fluorescent molecules in optical microscopy in images that include underlying clutter.^42,43^ The tracking method is based on (*a*) a detection and segmentation algorithm that localizes the particles retaining their image features into a particle probability image (PPI), and (*b*) correlating their image features to detect their tracks.^42–44^ If tissue motion is not a problem, there is no limit to the duration of the video loop that this algorithm can process, and the longer the video loop, the more tracks will be detected. Tracks are formed by linking detected particles so their number depends on the number of detected particles per frame, which is assumed to be fixed per frame. Wilson et al^43^ showed good performance in images with low signal-to-noise ratio (SNR) in microscopy applications; these images contain items similar to CEUS level of noise. In this work, image sequences of approximately 2000 frames (30 frames per second) were processed, and over 4000 tracks were created (SNR, ~2 dB) from 25,000 detected particles. These image conditions resemble CEUS images with a sparse MB distribution. Thus, this method can be applied to CEUS. The model-based approach allows the inclusion of prior knowledge on the evolution of MB signals^45–50^ that may lead to more efficient tracking. Emphasis is given to the detection and segmentation. This is because, unlike optical microscopy, ultrasound images have a highly variable PSF (ie, an individual MB will have a different appearance in different locations within the image), which is a significant challenge for the detection process. Methodologically, a ground truth is required for testing the algorithms, and this is provided by simulated environments. To date, super-resolution ultrasound work provides little information on localization accuracy and particularly on the role of detection and segmentation in this. Most experiments use vessel environments that do not provide this information,^23,25,51,52^ but rather demonstrate that the vessels can be resolved and that the MBs remain within the vascular space. The most common measure of such an accuracy is the full-width at half maximum (FWHM) of the diameter of each vessel^24,31^ or the size of a single MB PSF.^30^ Here, the detection and localization accuracy are assessed using synthetic data that simulates real data from animal or human studies. The accurate assessment of the number of detected particles by this approach, including spurious and missed detections, and the localization uncertainty are important for both methodology development and measurement of performance. As a second step, an in vitro vascular phantom validation is required. These methods do not replace the synthetic data validation above as no information on MB location, other than the fact that they are located inside a vessel, is provided. Instead, the aim is to assess image resolution. In addition, and by using real imaging equipment at settings that are relevant to clinical imaging, a convincing case is presented that MBs are detected and tracked as they flow inside microvessels with a resolution beyond the diffraction limit. A well-controlled experimental setup with a translucent phantom was used^32^ here.

However, such a setup has low variations of speed of sound compared with those generated by tissue. Thus, this idealized setup provides lower aberration in the near field of the array and the PSF of the entire setup is less variable compared with clinical imaging. This presents less challenging conditions to the operation of the detection, localization, and tracking methodologies. For these reasons, we chose to use an in vivo validation by means of optical coherence tomography (OCT). This ensures a live in vivo validation with exactly the same equipment, imaging parameters, and contrast agent, with real vessels and tissue aberration. The animal model used is the ovine ovarian corpus luteum (CL), a highly vascularized gland, which has previously been shown to be suited for perfusion imaging studies.^53^ Two further ex vivo methods for the microscopic examination of tissue, namely, optical microscopy and optical projection tomography (OPT), have been deployed to identify vascular features across the entire tissue that compare well with the ultrasound images. Finally, a proof of concept examination of a prostate patient is presented alongside the ex vivo histological evaluation for comparison. Imaging tumor vascularization is an active research field,^21,54–58^ including prostate tumors.^59,60^ The study of prostate cancer diagnosis includes all imaging modalities, and MRI seems as the most promising.^61^

## MATERIALS AND METHODS

The ultrasound image analysis was developed using synthetic, in vitro, and in vivo animal data. The latter used the sheep ovary and had 2 purposes: first, to provide the basis for generating synthetic data with a good approximation to in vivo image data; second, to validate the application of the methodology in vivo for clinically realistic ultrasound imaging parameters.

### Ultrasound Imaging

#### Animal Contrast Ultrasound Imaging

All animal data were collected under UK Home Office license approval (Duncan PPL 60/4401), and all the methods have been previously described.^53^ For each experiment, a female adult sheep was anesthetized, and each ovary in turn was exposed by laparotomy. The ovary was secured in place while maintaining full blood supply and was covered with a coupling ultrasound gel to achieve optimal contact with the ultrasound probe.^53^ This ensured that its shape was not distorted due to breathing motion and during imaging. Off-plane movements were minimized by using a reticulated arm that fixed the probe in a position where the scan plane was parallel to the breathing motion.

The methodology developed here is intended for use with standard CEUS that is currently available in the clinic. The contrast administration protocols were thus designed to provide a high density of single MB scattering events in the image, aiming to reduce the duration (eg, a few minutes), similar to that of a clinical patient examination. An iU22 Philips ultrasound scanner (Philips Medical Systems, Bothell, WA) with an L9–3 linear array probe (3 MHz) was used throughout (λ = 514 μm). Two contrast administration protocols were used. A 1.2-mL bolus of SonoVue (Bracco, Geneva, Switzerland) contrast agent and an infusion of 1.2 mL of SonoVue in 20 mL of saline at 0.5 to 1.5 mL/min rates using a Vueject pump (Bracco SpA, Geneva, Switzerland). All video data were saved in the DICOM format for offline processing.

#### In Vitro Imaging

To assess the performance of the super resolution image analysis in vitro, a cellulose tube, extracted from a single use renal dialysis filter (Dialyzer GFE-09, Gambro, Germany), secured to a 28-gauge microfill needle (World Precision Instruments, Stevenage, United Kingdom) with Luer connection was mounted in a Perspex tank. The tank was filled with boiled and cooled water. This setup was based on previously described in vitro work.^47^ The cellulose tube has a nominal internal diameter of 200 μm. Subsequently, and upon inspection using bright field microscopy, it was found to have a maximum internal diameter of 300 μm. The same ultrasound scanner and transducer as described previously were used. The transducer was mounted above the tube. 1.2 mL SonoVue contrast agent was added to 50 mL of water and allowed to flow by gravity feed through the cellulose tube. The end of the tube was placed out with the Perspex tank so no MBs entered the surrounding water. B-mode and contrast mode video loops of MBs flowing through the tube were acquired, saved, and later processed offline.

### Tissue Processing and Microscopy Imaging

To obtain information on the vascular structure of the scanned ovaries, 3 different tissue imaging methods were used: standard optical microscopy, OPT, and OCT. The OCT was undertaken in vivo after CEUS images were acquired. For microscopy and OPT, the ovary was removed after CEUS and processed as described later. As the only live validation method, OCT was expected to provide the most accurate data on vessel diameter and structure. This validation method serves a dual purpose: (*a*) it advances on an in vitro validation because the in vivo setup here provides the additional challenge of real tissue imaging aberration due to the variable speed of sound provided, and (*b*) it can be used as the best criterion standard for comparison with the other 2 optical methods that are performed ex vivo. Note that it is very difficult to provide MB location data using an optical validation method, so this task is undertaken by using the synthetic data.

#### Optical Coherence Tomography

Live images of the structure and information on the dynamics of the vascular bed were acquired using an OCT with a Telesto-II (wavelength 1300 nm, 5.5-μm axial resolution in air; Thorlabs, Lübeck, Germany).^62^ The OCT probe was secured directly over the top of the ovary and perpendicular to the ultrasound probe. This arrangement aimed at capturing the same image plane with both methods. This was a challenging experimental setting as the ultrasound probe had to be horizontal, imaging the top 1 to 2 mm of the tissue. Apart from the suboptimal gel coupling, the positioning of the ultrasound probe was not informed by the live OCT image, as the microvessels that appeared in the OCT are impossible to visualize in B-mode. The OCT data comprised a 3-dimensional (3D) data set of the light scattered from the tissue and blood at the surface of the ovary. Optical coherence tomography files were saved in ThorImageOCT software (Thorlabs, Lübeck, Germany) and exported to ImageJ for analysis.^63,64^

#### Light Microscopy

For ovaries undergoing histological slicing and staining, the process has been previously described.^53^ The removed ovaries were fixed, dehydrated, and embedded in paraffin, after which 5-μm-thick sections were sliced. For each 10th section, biotinylated lectin BS-1 (Sigma, United Kingdom), used to specifically bind to endothelial cells, was applied overnight at 4°C in a humidified chamber. Impact diaminobenzidine (Vector Labs, United Kingdom) was used to stain the endothelial cells. Sections were assessed by light microscopy (Olympus BH2) and a digital camera; images were saved at ×20 magnification. Vessels across the whole plane could be measured and counted. For comparison with ultrasound imaging, the slice taken from a location as close as possible to the imaging plane was chosen.

#### Optical Projection Tomography

Previous work formed the basis of the method here.^65^ Before removal of the ovary, 70-μL rhodamine-labeled *Griffonia* (*Banderiaea*) *simplicifolia* lectin 1 (GSL 1 lectin; Vector Labs, United Kingdom) in 7 mL of phosphate-buffered saline was flushed through the ovary followed by 7 mL of phosphate-buffered saline, using the main feeding artery of the ovary as the input source. The GSL 1 lectin was used to stain the main vessel endothelial cell walls. The ovary was then removed and stored in 4% paraformaldehyde. Before OPT, the ovary was immersed in 1 part benzyl alcohol (Sigma Aldrich, United Kingdom) and 2 parts benzyl benzoate solution (Acros Organics, United Kingdom) for 2 to 3 days to achieve tissue translucency. A Bioptonics 3001 OPT scanner (Bioptonics, Edinburgh, United Kingdom) was used to acquire tomographic scans generating 801 image files per scan at a rotation of 0.45 degree. Acquired images were 1024 × 1024 pixels with pixel size of 18.28 μm. The ovary tissue was autofluorescent in the green channel, and the GSL 1 lectin staining was visible on the red channel. Scan planes were combined to form a 3D reconstruction of the ovary using NRecon software (Skyscan, Kontich, Belgium). Interrogation of the 3D volume to select the same plane as that of the ultrasound image plane was undertaken in Bioptonics 3D Volviewer and in ImageJ (NIH.gov, United States).

### Synthetic Data Formation

#### Vessel Network

The first step was to simulate the MB movement within a vessel network. The simulation provided the spatial coordinates and identity of each particle at any time. This information was used as the ground truth for testing the subsequent localization. Such ground truth information is not available in in vitro or in vivo experiments. The synthetic vessel network consisted of a 1.1 × 2.2 cm grid of interconnecting vessels with diameters that ranged from 10 to 500 μm, while their length ranged from 25 μm to 2 mm. The particle flow within that network followed Poiseuille's law: by applying first a constant global pressure drop across the network and second the mass conservation law, the pressure field could be calculated. When the calculated elemental pressures were substituted in Poiseuille's law, the volumetric flow rate within each vessel was obtained. The flow simulation method is illustrated by a set of diagrams that are included in Boujelben et al.^66^ The injected dimensionless particles moved according to the calculated flow value.

#### Ultrasound Image Simulation

**FIGURE 1:**
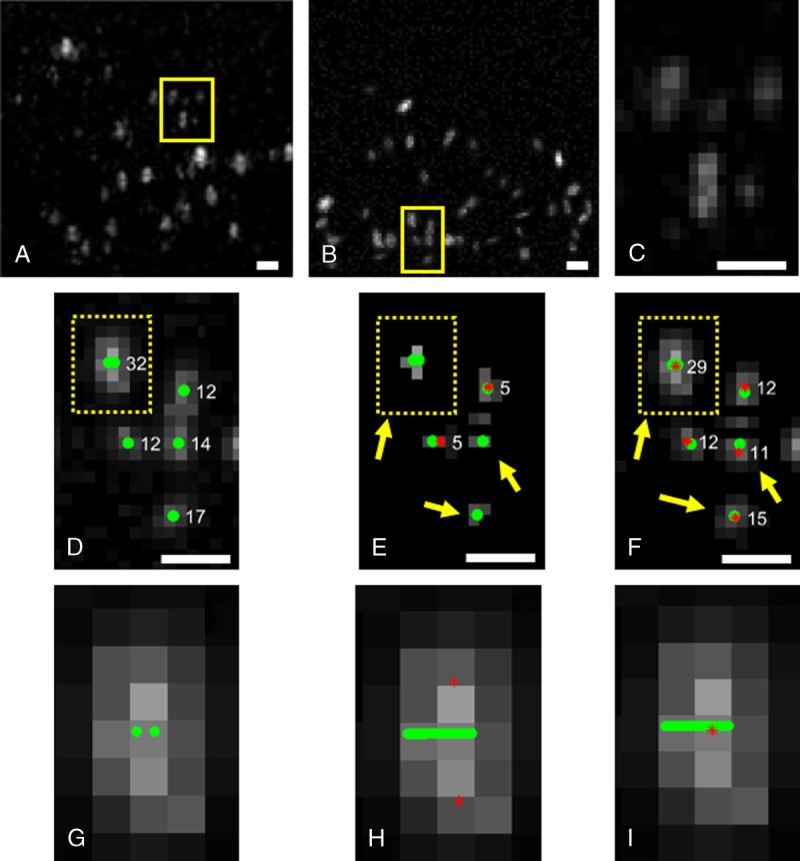
A frame (A) from a sheep ovary video loop and (B) from a synthetic data set constructed in resemblance with the previous in vivo data set. C and D, Images are the magnified yellow image sections from panels A and B, respectively. The final MB segmentation using panel E, the gradient image, and panel F, the inverted Gaussian image, as the input variable of the watershed transform. G, A magnified overlapping MB from panel D consisting of 2 single events. H, Detection of 2 single MBs that are located 172 μm and 224 μm away from the tube (the yellow line). Detection of one overlapping MB event that is located 14 μm away. The original location of the ground truth centers is in green circles, the detected MB centers by the software in red stars and their size in white label. Scale bars, 1 mm.

**FIGURE 2:**
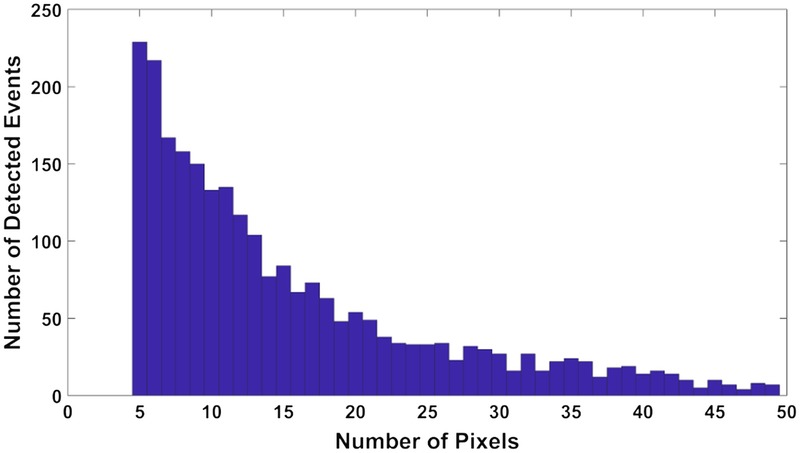
Histogram of the size distribution of the detected MB events. Pixel size is 132 μm.

The next step was to assign an MB echo to each particle position. It is known that the PSF is variable across the ultrasound image. As MBs are nonlinear scatterers of sound, they will provide echoes that are highly variable and difficult to model. To our knowledge, there is no research or commercial ultrasound image simulator that provides a reasonable MB echo simulation. Thus, we avoid a full calibration of the PSF here and instead generate a distribution of MB echo characteristics from in vivo video loops drawn from the sheep ovary experiment. A common characteristic of the MB echo image in this experiment was the ellipsoid shape of variable orientation (Fig. 1A). Three-dimensional Gaussian Fitting (minor, major axis, and intensity information) was used to extract the topology profile of the MBs in the in vivo data set. The resulting distribution provides a range of sizes, intensities, and shapes for MB echoes. This process can also be seen as an approximate estimation of the PSF. Note that it is accepted that the axial resolution is at best half the pulse length,^67^ which in our case is 1 λ (514 μm), whereas the lateral resolution varies across the image depending on the beam width^67,68^ and can be up to a few λ using the delay-and-sum beamfomers,^69^ which are used here. The histogram of the MB echo size distribution from the sheep ovary is displayed in Figure 2. The first and third quantiles are 8 (0.14 mm^2^) and 20 (0.35 mm^2^) pixels, respectively, whereas the median is at 12 pixels (0.21 mm^2^). Single MB echoes are unlikely to have sizes above 12 pixels (0.21 mm^2^), and the minimum detected MB is 5 pixels (0.09 mm^2^, approximately slightly larger than 2 × 2 pixels or one half λ × one half λ). Note that once 2 particles approach closer than a few wavelengths, they are likely to become larger in size before appearing as a merged particle in the image.^69^

In addition, the noise was characterized in the background and added to the simulated system as white Gaussian noise. This is because Rayleigh noise becomes additive Fisher-Tippet noise after log-transformation,^70^ which is well approximated by additive white Gaussian noise.^71^ Two hundred synthetic frames with simulated MBs were created. Figure 1B shows the result of the above process compared with a frame from a video loop of sheep ovary (Fig. 1A). Figure 1, C and D are the magnified squares from Figure 1, A and B, respectively, for further detail.

### Super-Resolution Image Analysis

An MB tracking algorithm was developed here to process the ultrasound video loops. The algorithm structure was based on a single particle tracking software previously used in optical microscopy.^43^ This software follows four main steps: (*a*) to detect particles, (*b*) to segment them (ie, identify their region), (*c*) to localize them (ie, find their center or exact location), and (*d*) to track them as they move from frame to frame. This process ensures that a highly accurate particle location is identified (super-resolution localization) and then its path is generated by joining particle locations at consecutive frames. The result can be displayed in particle density, path density, or velocity maps that contain different structural and dynamic information. Here, the particles are MBs that move only within the lumen of blood vessels. Thus, their localization is a location inside a vessel, and their path identification is equivalent to drawing the vessel structure. Because ultrasound imaging has different and highly variable aberration compared with optical microscopy, its PSF is highly variable. This results in particle appearance that varies across the image. Thus, the detection and segmentation part of the algorithm is central to the development here to suit the ultrasound MB/particle application.

#### Preprocessing

The CEUS images were processed to remove artifacts, such as bright echoes that were identified in the preinjection images, and were cropped from the entire frame sequence using ImageJ. Further, as off-plane movements were kept to a minimum, the well-established rigid registration^7^ was used to remove in-plane movement due to respiration. This was done using B-mode image features at the end-expiratory phase as previously described.^72^

#### Microbubble Detection

A semiautomatic detection process was designed to detect image particles with a range of sizes using an adaptive nonlocal means filter,^44^ which has shown to work well with super-resolution ultrasound microvessel imaging.^73^ The manual input of 3 parameters was required: the average MB echo intensity, the minimum and the maximum MB echo size. These are roughly estimated in an initial observation of the video loop. The MB intensity was preserved by using the PPI,^43^ a nonlocal mapping of the original grayscale image through the use of multiscale Haar-like features.^44^ These features were obtained after the convolution of 3 kernels, with variable formation and size that was determined manually according to the range of the particle sizes. The resulting Haar feature image is the linear combination of the maximum value of each spatial scale at each pixel. In the normalized Haar feature image, the pixels with higher values considered as statistically significant, which means they are likely to belong to an MB. A noise threshold is implemented in this image, which is the average noise level of the original frame sequence. This results in a binary image that is used to calculate the PPI. Subsequently, the particle probability in each pixel is the ratio of the number of pixels that have value equal to one in the binary image divided by the total number of pixels of the particle area.

Furthermore, a region threshold of 1/e of the normalized PPI is applied to initially estimate the target regions.^43^ This enables the discrimination of the foreground and the background and generates an initial segmented image. In parallel, the input image is convolved with a Gaussian kernel to create a Gaussian smoothed image, which is used to extract the local maxima of the image. Only the local maxima points that belong to a segmented region and have particle probability value above the threshold are preserved creating the final watershed seed points. These points determine the regions that the final segmentation process is based on. The final detection is refined by an automatic update in the last step of the detection process. Microbubble echo regions with a size below the minimum input size and input average intensity and above the maximum input size, which depended on the SNR of the frame sequence, were eliminated. This serves as a third noise classifier as all particles below the minimum size and average intensity effectively are considered as noise. This process also enabled the automatic MB detection update from frame to frame.

#### Segmentation

Segmentation is the key step to accurate localization. Marker-controlled watershed segmentation, as described, works efficiently in optical microscopy, where the PSF of the system is known and is generally symmetric and fairly constant in a frame sequence. In live-cell imaging, the algorithm implements segmentation by using a dilated gradient image of the Gaussian smoothed image. This image and the gradient image are the input values of the watershed transform. Subsequently, the watershed transforms segments of each particle. The change in particle area that is due to the dilation process does not affect the localization accuracy as the intensity-weighted center of mass method works well in a circular and constant PSF.

However, in CEUS, the PSF is variable not only between different frames but also within the same frame as the acoustic field and aberrations change across the image. As a result, MB echoes have nonregular shapes, which make the accurate segmentation of each region essential for efficient localization. Thus, the gradient image was replaced by an inverted Gaussian as the input variable of the watershed transform. The latter can better recover the MB echo area and avoid the reduction caused by the gradient image. This proved particularly useful for MBs that have low intensity and thus low SNR as well as overlapping MBs that are close to each other and were difficult to discriminate.

As mentioned previously, overlapping MBs are a significant number of detected events in the ultrasound imaging. By including these in the processing, data usage is maximized and it is possible to generate maps with short video loop duration. In super-resolution ultrasound, often a number of MBs situated close to each other, creating a large common echo, were mostly eliminated, and only clearly discriminated single (individual) MBs were included in the detection process.^2,3,24,25,30–32,35,52^ In optical microscopy, the accurate knowledge of the PSF permits the differentiation of particles that partially overlap in the image,^43,74^ and all the particles are possible to localize. This is difficult to implement in CEUS images due to the lack of PSF constancy.

However, it is possible to treat overlapping MB events as single. The rationale is that a single center has high likelihood to be located inside its vessel. The overlapping MBs may follow the same detection and segmentation process as a single event. Thus, the maximum MB size is set to a large value to process regions of both single and overlapping MBs. The algorithm can determine the size and the neighborhood of the detected MBs by adjusting 2 parameters, the Gaussian smoothing and the local maxima width. Fewer detections may occur if either the Gaussian smoothing or the width are increased. Because in an overlapping event there may be low confidence as to whether the local maxima truly represent single MBs, the choice of larger values in the Gaussian width and the local maxima width enables a single detection. Finally, overlapping events follow the same process of the birth and death of single MBs within the algorithm, permitting the split and merge of the events,^42^ which may occur in consecutive frames.

#### Microbubble Localization and Tracking

The final segmented regions (MBs) were used for the localization of each MB. The intensity-weighted center of mass^43^ that is commonly used in ultrasound field^24,30,33^ was also used here. This is because the final segmented regions preserve all the original information to deploy this method. Alternative methods include the use the FWHM,^25^ a deconvolution of the PSF,^23,26,31^ or the local maxima to calculate the center of the PSF. These methods do not deploy the intensity of the segmented regions or rely heavily on the assumption that the PSF is constant and were not the first choice here. Microbubble linking in consecutive frames (ie, tracking) was completed using the nearest neighbor method.^42^

Following the linking process, all the tracks were superimposed in one figure, creating this way the final density map. The pixel value in this map was the number of tracks that passed through this pixel. Further, this algorithm provided information about the speed of the MBs, and an MB velocity map was generated, which represented the mean velocity of the tracks that passed through each pixel. The pixel size can be at the original size or at a subdivision of the original size reflecting the localization uncertainty. Through synthetic data experiment, the level of the subdivision was determined by creating a subpixel similar to the root mean square error (RMSE) of the localization uncertainty.

### Performance Metrics/Criteria

#### Synthetic Data

The detection efficiency and localization accuracy of the algorithm was tested by means of five statistical measurements on the synthetic data as commonly used in optical microscopy.^75^ These are as follows:

- *Root Mean Square Error*. The RMSE evaluates the localization accuracy of the software using paired particle events. Each ground truth event is paired with the result of the algorithm that is in its vicinity. The distance between the 2 provides a measure of the localization deviation distance. For the number of pairs in the image sequence, this is given by the following formula:

**Formula:**
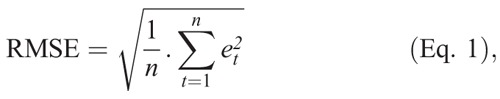


where *e_t_* is the localization deviation distance for the pair *t*. Thus, the lower the RMSE value, the better the localization.

- *Missed Events*. Any ground truth event that does not pair with a detection event is counted as a missed event.
- *Spurious Events*. Any detected event that has not been attributed to a ground truth event is counted as spurious events.
- *Minimum Distance*. This is the minimum localization deviation among paired events.
- *Maximum Distance*. This is the maximum localization deviation among paired events.

#### In Vitro Data

The algorithm performance was tested by comparing the resulting vessel diameters on the final density maps with the diameter of the tube.

#### In Vivo Data

The accuracy and efficiency of the methodology was evaluated by comparing features on the final density maps with those identifiable on optical microscopy, OPT, or OCT. Cross-section area measurements from the CL and the follicles as well as vessel diameters from arteries, arterioles, and follicle wall vessel diameters were used.

### Proof of Principle Prostate Patient Data

The method was also tested in vivo for the ability to resolve the prostate microvasculature in a patient referred for radical prostatectomy at the AMC University Hospital (Amsterdam, the Netherlands) because of biopsy-proven prostate cancer. An intravenous peripheral injection of a 2.4-mL SonoVue bolus was performed. The bolus passage through the prostate was recorded for over 2 minutes by transrectal ultrasound imaging with an iU22 ultrasound scanner (Philips Healthcare, Bothell, WA) equipped with a transrectal probe C10-3v. For the acquisition, a power modulation pulse scheme at 3.5 MHz was adopted to achieve contrast-specific imaging, and the mechanical index was set equal to 0.06 to avoid bubble destruction. The frame rate was equal to 10 Hz. The probe was held at a fixed position, and no breathing movement was observed. After radical prostatectomy, the resected prostate was first fixated and then cut in slices of 4-mm thickness, which were then marked by a pathologist to delineate prostate cancer based on microscopic analysis of cellular differentiation.

## RESULTS

### Development of the Super-Resolution Algorithm on Synthetic Data

A real-frame sequence from a sheep ovary was used to generate a sequence of synthetic ultrasound frames. Typical frames are shown in Figure 1, A and B, respectively.

Figure 1, D to F illustrate the result of the new segmentation process. The original synthetic image (Fig. 1D) was segmented using the watershed function. The result from implementing 2 different methods, the gradient image, as used in optical microscopy (Fig. 1E), and the inverted Gaussian (Fig. 1F) are shown. As a first observation, the size of the PSF of the segmented MBs were smaller (number of segmented pixels displayed next to each MB in Figure 1D) when the gradient image was used (Fig. 1E) compared with that of the inverted Gaussian (Fig. 1F), which had very good agreement with the original data (Fig. 1D). This slight difference between these 2 sets of measurements (Fig. 1, D and F) is based on the existence of the noise in Figure 1D and the manual calculation of the size of each MB there, whereas in Figure 1F, the same calculation took place after the process of noise removal by the algorithm.

Table 1 summarizes the difference between the 2 segmentation approaches. Better size and shape recovery (Fig. 1F) of the original MBs (Fig. 1D) led to the significant improvement in localization accuracy. The RMSE was 3 times lower using the inverted Gaussian (25.8 μm) compared with the gradient image (78.6 μm). Second, there was a significant decrease of the missed events using the inverted Gaussian method to 0.07% (Table 1). The high occurrence of missed events using the Gradient image is explained by the removal of scatterers by the algorithm, which was significantly reduced in size using the gradient image (arrow in Fig. 1, E and F) as they have size below the MB echo input size. As a result in Figure 1E, 2 of 5 detections are recorded, whereas in Figure 1F, 5 of 5.

**TABLE 1:**
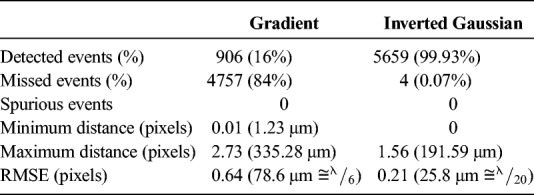
Statistical Results for the Detection and Localization Accuracy on Synthetic Ultrasound Data (5663 Single MB Events in 200 Frames) Using the Gradient and the Inverted Gaussian Image as the Input Variable of the Watershed Transform

**FIGURE 5:**
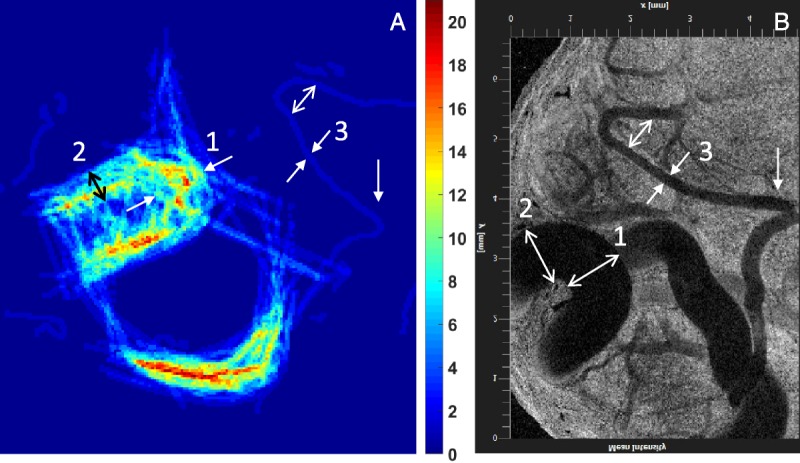
Comparison of panel A density map obtained from a SonoVue infusion at 1.5 mL/min (936 frames) with panel B OCT mean variance image at depth comparable to that of the ultrasound scan. The ultrasound transducer was placed perpendicular to the OCT probe and as close as possible to the surface of the ovary while maintaining contact for imaging. A Thorlabs Telesto-II OCT machine (wavelength 1300 nm, 5.5 μm axial resolution in air) provided clear images on the vessels closest to the surface of the ovary. The arrows indicate comparable locations with example measurements from OCT being location 1 0.98 ± 0.01 mm, location 2 0.96 ± 0.02 mm, and location 3 228 ± 23 μm compared with measurements from the density map of 1.20 ± 0.01 mm, 1.10 ± 0.02 mm, and 236 ± 15 μm, respectively.

Overlapping events where MBs were merged in appearance in the synthetic data were assessed. An example is shown in Figure 1G where 2 MBs are 59 μm apart. If the algorithm is used to deploy the detection of local maxima to identify each of the 2 MBs (Fig. 1G), they are (Fig. 1H) 396 μm apart, and 172 μm and 224 μm away from the tube, respectively. Increasing the typical particle size that reduces the segmented regions, the 2 merged MBs were treated like a single event (Fig. 1I). The distance of this event from the center of the tube was 14 μm. The processed synthetic video loop contains 10,780 single MBs in 200 frames, which resulted in 2442 overlapping MBs (mostly due to double particle overlap) and 5896 well-separated single MBs (8338 in total). The overlapping events were effectively 2 or more MBs that were in a distance below the three fourth of the wavelength (385 μm) from each other. These were treated as single events. The number of both single and overlapping events is in a good correspondence with the software result that detected 8117 events.

### Assessment of Super-Resolution Methodology in Real Imaging

#### Performance Assessment In Vitro

**FIGURE 3:**
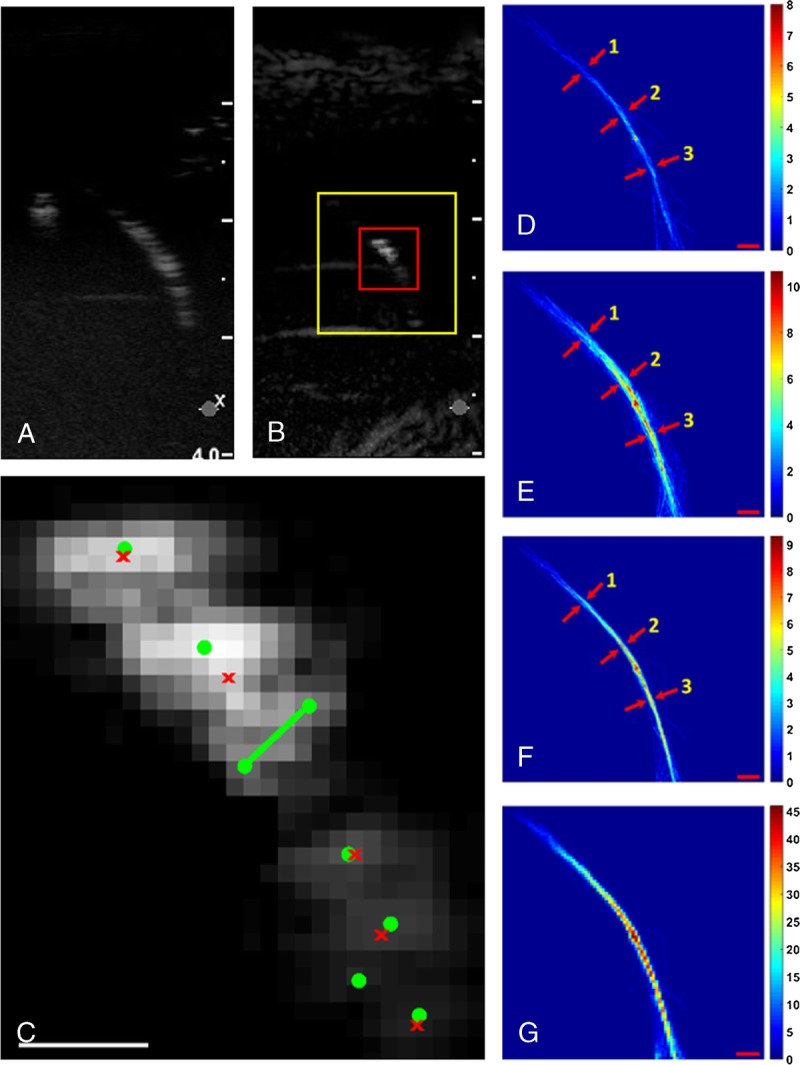
A, A B-Mode frame and (B) the corresponding contrast mode frame from a single tube in an in vitro experiment. C, Localization example within the red box (A) trying to detect single events (green circles) and single and overlapping events (red crosses). Trying to segment the region into single events creates localization 678.48 μm apart (green line), which implies localizations outside the tube (tube width is 200–300 μm). The density map with 3 × 3 subdivision process as the result of the tracking algorithm using the inverted Gaussian image as input of the watershed function detecting (D) only (isolated) single MBs, (E) any (segmented) single MB, and (F) both single and overlapping MBs. G, The density map of (E) in original pixel size. The FWHM was calculated in 3 parts of the tube, 1–3 and as it shown in panels D, E, and F pointed with red arrows and explained in details in Table 2. The scale bar is 1 mm throughout.

The results for the in vitro feasibility test are shown in Figure 3. The video loop comprised 300 frames and was collected using the same ultrasound settings as the in vivo work. A B-mode frame and the corresponding contrast mode frame are displayed in Figure 3, A and B, respectively. The methodology using the inverted Gaussian image was used to process the in vitro frame sequence. This was limited to an ROI that was away from artifacts (yellow box of Fig. 3B). Figure 3C presents an example processing with detections within the red box of Figure 3B. The events that result from segmenting overlapping events into 2 or more single ones (green circles) and those that result from treating overlapping events as single (red crosses) are presented. It can be seen that in treating overlapping events as single leads to localizations inside the tube while segmenting these into single events leads to 2 localizations that are 600 μm apart (tube maximum internal diameter, 300 μm). Thus, segmenting overlapping events is often erroneous and leads to an overestimation of vessel size.^69^ Further, the density maps at a 3 × 3 subdivision of the original pixel size were studied using the 3 available detection processes. Figure 3D shows the density map that resulted from detections of only single and well-separated events, whereas Figure 3E used single events that stem after the segmentation of overlapping events, and Figure 3F detects overlapping events as single along with the well-separated single ones. The FWHM at the same 3 example positions (1, 2 and 3) was measured for each of the above processes and are given in Table 2. This shows that the diameter estimation is best depicted by the map of Figure 3F, where overlapping events are treated as single. Note that the lack of data in Figure 3D leads to an underestimation of the diameter and the lack of accurate PSF knowledge leads to an overestimation of the diameter in Figure 3F. Finally, Figure 3H shows the corresponding density map for Figure 3E using the original pixel size.

**TABLE 2:**
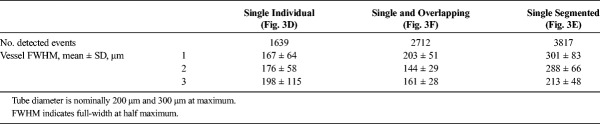
Number of Detected Events In Vitro and FWHM of the Tube Following Different Processing (Fig. 3)

The vessel diameter is depicted accurately when overlapping MBs are treated as single and confirms our hypothesis on the overlapping MB location. These results are in agreement with the synthetic data. By treating overlapping events as a single event, all localizations are found inside the vessel, and the vessel width is depicted accurately. On the other hand, our knowledge on the PSF is not adequate to inform the splitting of overlapping events accurately (similar to Fig. 1H), and therefore they can be located outside the vessel resulting in an overestimation of the vessel width (Fig. 3E). In addition, when only well-isolated single MBs are used, the number of detections drops by 40%, compared with when overlapping events are accounted for (Table 2), which leads to an underestimation of the vessel diameter.

#### Algorithm Performance Assessment In Vivo

The extracted ultrasound video loops comprised 500 to 1500 (processed) frames with frame rate of 12 to 13 Hz. All videos were acquired in the wash-in or the wash-out period of a bolus injection apart from the videos used for OCT comparison, which were generated by using an infusion of sparse MBs. The mean video duration for bolus injections was 121 ± 19 seconds and for infusions 92 ± 28 seconds.

**FIGURE 4:**
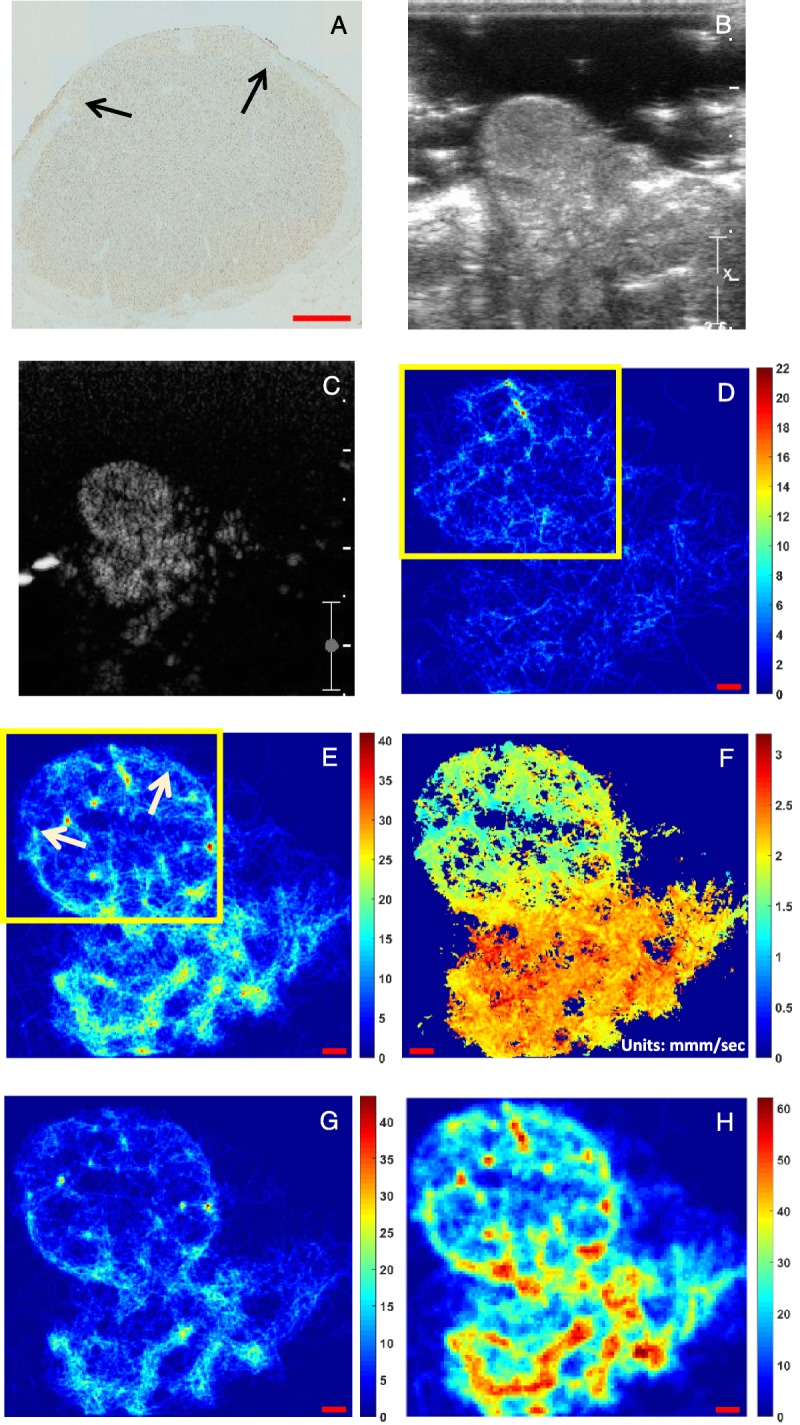
A, Histology (×20 tiled microscopy) of the corpus luteum (CL) of a sheep ovary. B, The B-mode frame and (C) the corresponding peak contrast frame from the CL include the supporting artery and vein branches from the ovarian artery and vein respectively that support the gland and are not available in (A). The density map with 3 × 3 subdivision process as the result of the tracking algorithm using the inverted Gaussian image as input of the watershed function and detecting (D) only single MBs and (E) both single and overlapping MBs while (F) is the corresponding velocity map for (E). Panel G is the equivalent of E using the gradient image instead of the inverted Gaussian. Panel H is the equivalent of E in original pixel size. The color bars indicate the number of the tracks per pixel for the density maps and the average mean velocity (millimeter per second) for the velocity maps. The scale bar is 1 mm throughout.

Figure 4 illustrates an example of the algorithm performance in an in vivo setting depicting the density maps using the original pixel size (132 μm) or the 3 × 3 subdivision of the original size (44 μm) as described previously. Figure 4A shows the tiled microscopy image, lectin stained to show endothelial cells, of a slice of a typical mature CL of the sheep ovary (day 9). The circular pattern is due to the near-spherical shape of the CL. This shape is the result of the rapid angiogenic growth of the CL in the first 9 to 10 days of the oestrous cycle of the ewe. Its vascular architecture here shows that the largest arterial feed surrounds the tissue and branches into smaller arterioles. The size of this artery is between 200 and 300 μm. The arrows in Figure 4A show that the artery is no longer in view in this slice, which is due to continuation and branching off plane. Branching of this artery into arterioles is evident across the entire length of these arteries. The core of the CL is mostly filled with microvessels. Not shown in Figure 4A is the supporting ovarian vessels (artery and vein) that lie under the location of the CL. Figure 4, B and C are the B-mode and peak contrast image in CEUS mode, respectively. The MB movement was tracked through the CL, 27 seconds after the peak contrast image and when the population of MBs was sparse enough for the algorithm to operate. The video loop consists of 583 frames, and its duration was 48.6 seconds. In Figure 4D, the processing includes the inverted Gaussian, and the parameters are adjusted for single MB detection only. The number of detections were 10,906, the average number of detections per frame was 19 (ranging from 1 to 42), and the number of tracks was 1135. Note that, in the absence of ground truth, the number of missed or spurious events cannot be quantified. Figure 4E displays the path density map that uses the inverted Gaussian in the segmentation process and is set to detect both single and overlapping MBs. This resulted in 34,325 detected events, which is on average 59 events per frame (ranging from 7 to 138), and 2951 tracks. The display has a pixel size 44 μm. Figure 4F shows the corresponding mean velocity map. Figure 4G shows the density map for single and overlapping MBs just as in Figure 4E, but uses the gradient image in the segmentation process instead of the inverted Gaussian. Finally, although Figure 4, D to G used the 3 × 3 subdivision process, the respective density map of Figure 4E with the original pixel size is shown in Figure 4H.

There is good resemblance between Figure 4A and Figure 4E, whereas Figures 4D and 3G depict a number of sparse tracks that bear little resemblance to the vascular architecture of the sheep ovary (Fig. 4A). Arrows in Figure 4E show a very similar location of the termination of the feeder arteries (arrows in Fig. 4A). Further, these larger vessels have a large number of paths (in yellow) compared with the inner CL, which agrees with their large comparative size shown in the histology. In addition, a number of smaller vessels are shown to branch inwards from these feeder vessels as shown in the histology slice. The rest of the inner CL area has a small number of paths that may be attributed to low microvessel flow. Under the boxed area of Figure 4E is the ovarian artery and vein, not shown in Figure 4A. These display the largest density of paths in Figure 4E and largest blood velocity (Fig. 4F). These features are not apparent in Figure 4D and Figure 4G that use only single MB processing and gradient image approach in the segmentation, respectively. In addition, using the original pixel size for localization in density maps (Fig. 4H) cannot depict different types of vessels. The feeder vessels that surround the CL (200 to 300 μm) and other microvessels inside the CL do not appear different to the ovarian artery and vein below the CL that are a couple of mm wide.

Density maps were obtained and processed similarly from 6 contrast video loops, which were typical of the range of data sets that were acquired, and the information is displayed in Table 3. From these, 1 was in wash-in and wash-out of the bolus, limiting the process only within the CL, 3 were in the wash-out and 2 were captured during an infusion of sparse MBs. The duration of each video loop varies from 39 to 129 seconds.

**TABLE 3:**
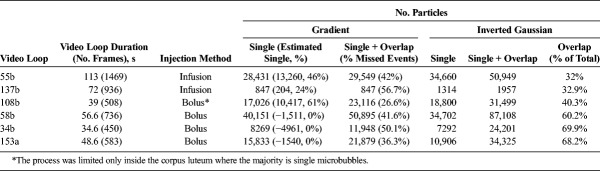
Number of Detected Events In Vivo

Table 3 shows that detecting both single and overlapping MBs using the new methodology with the inverted Gaussian as the input for the segmentation process maximized the number of detected events. As there is no ground truth in these data, a manual observation confirmed that the proportion of missed (undetected MBs) and spurious events (wrong detections) is low and did not affect the final density map. Indeed, within the CL, there is clear vascular network pattern. This table shows that the overlapping and single MBs using the inverted Gaussian (column 7) was always the maximum number of detected events in each data set and thus optimal detection. When the algorithm was adjusted to count the single events (column 6), it was shown that the overlapping events were between 32% and 68% of the total number of detected MB events. Columns 4 and 5 show the particle underestimation effect of using the gradient image as input for the watershed function instead. When both single and overlapping events were used in the detection process, the number of events accounted for were between 17% and 57% less than the ones that were counted using the inverted Gaussian. This was due to the shrinking of these particles' areas resulting from the gradient image calculation. The shrinking caused a lot of MB events to diminish to sizes that were not more than 5 pixels, which is the minimum input size threshold, and thus were eliminated. In addition, the size discrimination between single and overlapping events became more difficult to implement. This is because the algorithm size threshold was implemented after the segmentation process, and the shrinking due to the gradient image resulted in a lot of overlapping MB events to be misclassified as single. The resulting column 5 is likely to include mostly overlapping events, as many single ones were removed by the minimum input size threshold. In addition, when the particle size parameter was adjusted for single events, then the number further decreased, but a large number of overlapping events remained included (column 4). If it is assumed that the total number of true MB events is approximately that of column 7 and that the single events are reasonably approximated in column 6, it is then possible to produce an estimated number of single MB events accounted for in column 4, which is the difference between the column 5 number and the number of overlapping events (difference of columns 7 to column 6). This varies between 0% and 63% of the total number calculated. Negative numbers in brackets signifies that no single events are accounted for, and a number of overlapping events were also missed. These results confirm the synthetic data behavior observed in Figure 2, D and F.

#### Validation of In Vivo Ultrasound Size Measurements

Density maps from 10 different ovaries from 8 different sheep were compared with either histology (4 ovaries), OPT (6 ovaries), or OCT (2 ovaries) imaging. From these, 8 were bolus injections and 2 were captured during an infusion. Optical coherence tomography provided a live image of the vessels close to the surface of the ovary in situ, and the comparison with the respective density map is displayed in Figure 5. The main vessels are clearly seen in the density map (Fig. 5A) and the OCT image (Fig. 5B). Table 4 provides a summary of the measurements made. For the images shown in Figure 5, the vessels were measured to have diameters of 0.9 ± 0.01 mm from OCT compared with 1.19 ± 0.01 mm on the density map, and the narrow vessel diameter was measured to be 228 ± 23 μm from OCT compared with 236 ± 15 μm. The mean difference in measured sizes between the 2 was approximately 10%, which is not significant. Thus, OCT provided the best agreement between density maps, showing that the density map (Fig. 5A) can provide quantitative and accurate information of vascular structures, including vessel diameter and bending.

**TABLE 4:**
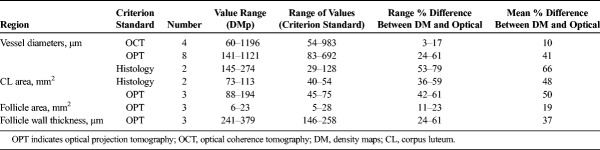
Measurements of Sheep Ovary Structures From Histology, OPT, and OCT Ultrasound Density Maps

**FIGURE 6:**
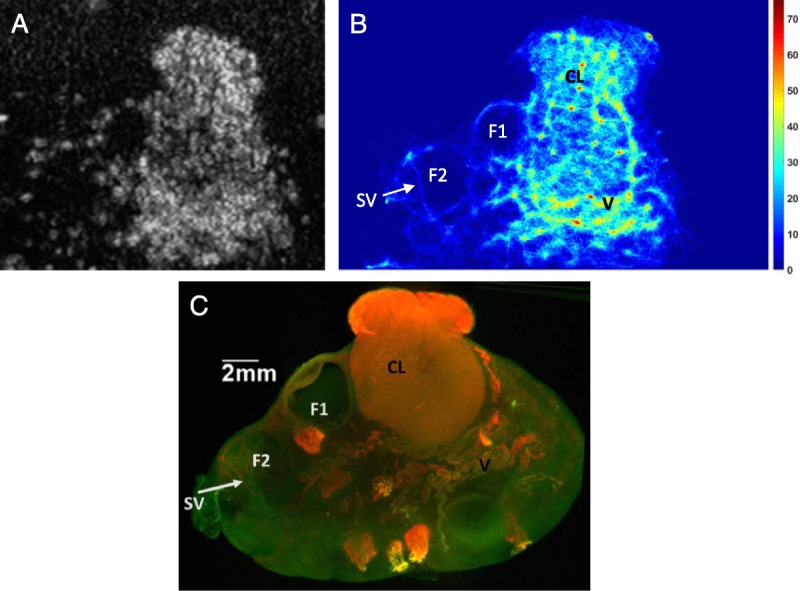
Comparison of a density map (by processing 1246 frames) obtained by imaging the sheep ovary with CEUS and using a 2.4-mL bolus injection of SonoVue, with a subvolume selected from a 3D reconstructed OPT volume (A) peak contrast ultrasound image, (B) density map, and (C) OPT image using maximum intensity projection to display a 2-mm-thick volume. There are structures in the ovary identifiable in both the density map and the OPT slice such as the follicles (F1 [OPT: area 5.6 mm^2^ wall thickness 173 μm; density map: area 6.4 mm^2^ wall thickness 344 μm], F2 [OPT: 11.3 mm^2^ wall thickness 258 μm; density map: area 14.7 mm^2^ wall thickness 340 μm]), the CL (OPT: area 45.3 mm^2^; density map: area 88 mm^2^), and larger vessels (V). The dip in the follicle 1 wall in the OPT image is due to the delicate surface of the follicle on the edge of the ovary collapsing during the processing. Arrowed is a small vessel (SV) located on the surface of the follicle that can be identified in the OPT image (83 ± 7 μm) and the density map (diameter 141 ± 54 μm).

Compared with the OCT, the OPT provided information from larger areas of the tissue and in 3D. Compared with standard optical microscopy, OPT provided a 3D reconstruction of the whole ovary. It was then possible to choose a subvolume at a region of choice, which could also have a similar thickness to the ultrasound scan plane, which was approximately 2 mm, for comparison. This is not possible with standard 2-dimensional (2D) microscopy where the slices have a thickness of approximately 5 μm. Their orientation is roughly estimated before slicing, and therefore the comparison with the ultrasound image is difficult to make. Thus, in the example of Figure 4, it is rather fortuitous that Figure 4A appears very similar to Figure 4E. An example of a density map and the corresponding OPT slice are shown in Figure 6. The structure of the ovary from the ultrasound image can be seen in each modality: (*a*) peak contrast in CEUS mode, (*b*) density map in super-resolution processing, and (*c*) OPT. There are structures in the ovary identifiable in both the density map and the OPT slice such as the follicles (F1, F2), the CL, and larger vessels (V). The 2 follicles are well defined by the density map, and the large CL has a dense vascular network. On the smaller scale in the density map, there is a track detected in the first follicle, which is assumed to be part of the outer surface of the follicle. This is also seen in the OPT image (arrowed). This vessel measures 141 ± 54 μm diameter on the density map compared with 83 ± 7 μm on the OPT.

The main comparison performed between density maps and histology slices was on the measurement of the area of the CL. For 3 density maps and histology pairs, the mean CL area measured 48% smaller on histology than that measured on the density map. For all measurements, the mean difference in sizes measured on OPT is 36% smaller than that measured on the density map. Of the measurements made on the density maps, the smallest measurable regions that could be compared with similar regions on OPT were small vessels and follicle walls. A vessel diameter measurement on OPT at 83 ± 7 μm is compared with 141 ± 54 μm on the density map. Follicle wall thickness was measured to be 173 ± 35 μm on OPT compared with 241 ± 10 μm on the density map. It is evident that histology and OPT provide a significant underestimation of all sizes and area measurements compared with the density maps.

Finding comparable vessels in the optical images that were smaller than 100 μm and could be compared with the same vessels in the density maps was challenging. This is because all the different criterion standard techniques did not provide an abundance of such measurements. The OPT staining was inadequate to delineate arterioles, whereas the OCT resolution and sensitivity are not adequate to show the smallest vessels. As mentioned previously, the 2D microscopy with lectin staining provides such vessels but cannot be matched with the ultrasound maps. A number of vessels below 100 μm were observed, however, in the ultrasound maps and are displayed in Table 5 but were not verified. However, the large number of tracks strongly suggests the existence of vessels. The narrowest vessel measured on the density maps was 55 ± 10 μm, and there are several clear vessel paths between 50 and 100 μm.

**TABLE 5:**
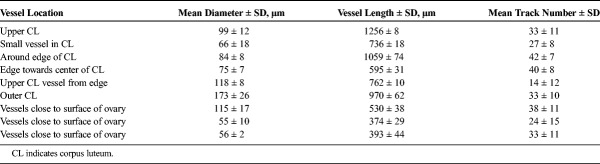
Unverified Ultrasound Density Map Measurements

#### Example Map of the Prostate

**FIGURE 7:**
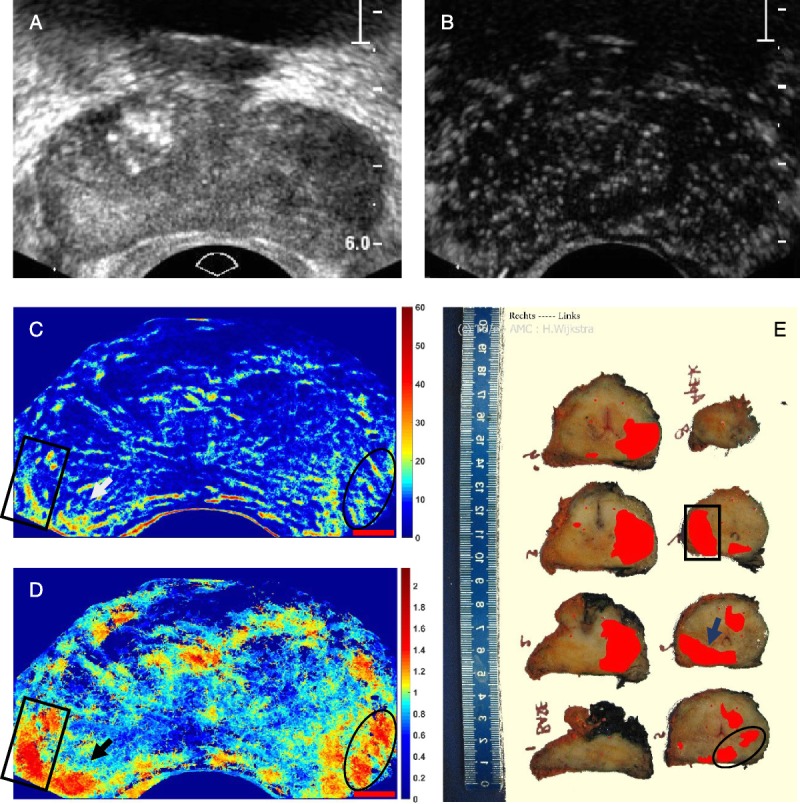
A, B-Mode and (B) contrast mode of a human prostate with a sparse MB population apparent in the late wash-out phase. C, Ultrasound path density map (color map shows number of MB paths). D, Ultrasound velocity map in millimeter per second. Red scale bars in C and D are 5 mm. E, Histology slices of the prostate displaying cancer in red.

The MB tracking algorithm was applied to a human prostate with cancer. Figure 7, A and B are the B-mode and a contrast image after the peak of the MB density in CEUS mode, respectively. The acquired data set was typical and of low SNR. The video duration was 136 seconds from which the 115 seconds was the processed time that provided a density and velocity map (Fig. 7). The detection parameter combination was optimized to detect both single and overlapping MBs. A total of 1149 frames were processed, where 612,439 events were detected and created 47,536 tracks. The examination time had to be kept as short as possible; hence, an MB bolus injection was used. As a result, there is high MB density per frame, where the average number of detections per frame was 533.

The marked areas by a rectangle shape, an oval shape, and an arrow in Figure 7, C and D show the tumor areas that correspond to the histology. In the histology (Fig. 7E), cancerous areas are displayed with red color, based on the microscopic analysis of cell differentiation (Gleason's pattern) of whole-mount sections according to Montironi et al.^76^ Before the histopathological analysis, the prostate was fixated in formalin for 24 hours and sectioned in 4-mm slices. Note that the histological slices and ultrasound imaging planes are not parallel, and one imaging plane can intercept multiple slices. The velocity map provides very good correspondence to the histology particularly slices 5, 6, and 7, whereas the density map provides also a reasonable agreement with these slices.

## DISCUSSION

Super-resolution images under realistic patient imaging conditions were achieved, demonstrating the feasibility of clinical 2D ultrasound super-resolution imaging using a standard CEUS mode. The gain in resolution is at least 5-fold, as vessels under 100 μm were detected at transmit frequency of 3 MHz (λ = 514 μm), and the system resolution here is approximately λ (half the pulse length). The smallest verified vessel width was 60 μm (Table 5), and the unverified detection of small arterioles (55 μm) presented nearly an order of magnitude resolution gain. The synthetic data investigation shows that the MB localization uncertainty can achieve 26 μm accuracy. The use of synthetic data enabled the development of the method into an ultrasound one as the errors induced by several parts of the processing were possible to assess and minimize. This was done by investigating detection efficiency, segmentation accuracy, and subsequently MB localization accuracy. The in vivo results are comparable with the literature in terms of resolution improvement. Experiments in thinned skull of rats in a fixed location provided λ/6 resolution at 20-MHz transmit frequency and using ultrafast scanning.^31^ Elsewhere, λ/4 resolution was achieved using higher transmit frequencies for identification of tumors.^33^ A clinical scanner has been used in the initial demonstration of super-resolution imaging in vivo, and under 20 μm resolution was achieved, which is over 5 times the improvement to the system resolution.^24^ This was performed in an optimal setting to minimize aberration, as only a thin slice of tissue was scanned, a flattened mouse ear, and the depth was also limited to 1 cm, and the tissue and probe were static. Our results that resolved structures with at least λ/8.5 accuracy were performed under conditions that are closer to clinical 2D CEUS using standard CEUS mode, low frame rate, radiology-relevant image depth to investigate a volume of tissue. Thus, compared with the above studies, significantly increased aberration was present in the in vivo data here. This results in additional PSF shape distortion with potentially negative consequences in MB detection and segmentation. Given the approximately 2-mm thickness of the 2D ultrasound plane, it seems surprising that vessels with tens of micrometers in diameter can be visualized. This is explained as the tracking algorithm enables the super-resolved MB localization across the third dimension of the slice thickness. The resulting density and velocity maps are in fact a projection of the ultrasound-exposed 3D volume into a 2D image plane. Thus, provided that scattering events are possible to distinguish, vessel structure is preserved projected and so is blood velocity. This is because different vessels may lie at different angles (ie, from vertical to parallel) in relation to the scan plane. This may result in inaccurate depiction of velocity as the angle is not known. However, in principle, 2D super-resolution imaging in vivo is not hampered by the width of the ultrasound scan slice, and several microvessels are possible to depict in density maps. Although it can be argued that in the future the velocity accuracy may be improved using 3D CEUS, the 2D prostate image here (Fig. 7D) strongly suggests that the velocity accuracy is not a problem as the high blood velocity were detected only around the tumor and correlated well with its area. This may be attributed to the high density of tumor neovessels that also have irregular pattern. This ensures that a large number of vessels are parallel to the scan plane. All this suggests that, in a clinical study, different types of velocity maps need to be tested to identify those that represent closest tumor dynamics in 2D (eg, maximum velocity that may be hypothesized to represent parallel vessel velocities). In addition, tissue motion artifacts do not appear in the super-resolution literature and seem to be well compensated here. Note that the animal experimental setup ensured that nonrigid motion is avoided and that the rigid motion due to breathing is kept in plane with the 2D ultrasound image plane. Thus, the well-established rigid registration provided good compensation. Further, the prostate did not require tissue motion compensation as breathing motion does not affect the position of the tissue.

The short video loop time (approximately 2 minutes) here, which provided adequate data for processing, strongly suggests that a clinically relevant examination time is feasible. However, such reduction of data results in additional challenges for the processing compared with other studies. The short video loop time required the use of a large number of MBs per frame. Table 3 shows the number of the detected events (single-overlap column) and the time for each processing, giving an overview of the average number of detections per frame. Comparing with literature, our algorithm detects, for the case of prostate cancer, 533 events on average per frame in 1149 frames, whereas the corresponding values, for example, in Errico et al,^31^ are approximately 13 events per frame, for a 75,000-frame data set. As mentioned previously, millimeter-sized vessels have orders of magnitude more blood volume, and thus MB concentration, compared with microvessels. At sparse MB infusion concentrations, this, in theory, results in very few single MBs in the microvascular bed, whereas a lot more and several overlapping MBs will appear in the larger vessels. Indeed, it was found here that more than 32% of detected events are attributed to overlapping MBs, which implies that overall these account for more than 50% of the MBs in the image, as shown in the CL study (Table 4). Previous investigations tend to avoid overlapping MB events.^24,31,32^ In this case, large data sets are required to depict the entire vascular space under investigation,^24,25,31,52^ which implies that clinical examination times would be significantly increased. The advantage of excluding overlapping events is that no assumptions are needed to include these events, and the localization accuracy is optimized. However, using only the single events the visualization of large vessels in their entirety may not be depicted accurately. The comparison between Figure 4D (single only) and Figure 4E (overlapping MBs included) showed that, within the approximately 49 seconds video loop time, the inclusion of only single MBs in the processing provided maps that do not include larger arterioles and feeder vessels. In other words, the exclusion of overlapping events provides a systematic error in mapping the vascular bed. The inclusion of overlapping MB echoes in the processing provided a more accurate depiction of the vascular structure with better representation of MB path proportion in different-sized vessels. It is suggested that the path density correlates well with volume flow, whereas the exclusion of overlapping MBs results in a blood volume estimation error for larger vessels. This further strongly suggests that the assumption that most overlapping events are likely to be located in the larger vessels is correct.

The challenge of including large MB numbers in the imaging may be best addressed using high frame rate imaging, which can provide MB scatter overlap and deploy tracking using the autocorrelation method.^31^ As mentioned in the introduction, such frame rates require a plane wave transmission that provides high attenuation and limited penetration. Further in CEUS, this limitation further increases the variability of the MB detection efficiency across the image due to increased S/N variability and MB destruction. Thus, the detected MB density and path density do not correlate well to red blood cell density and volume flow, respectively, which severely limits quantitative super-resolution maps of vascular dynamics. Here it is demonstrated that the less variable field of the focused transmission at low nondestructive acoustic pressures ensures reasonably uniform MB detection, with high penetration depth up to at least 6 cm (Fig. 7A). In addition, and as mentioned previously in Errico et al,^31^ an average of 13 events per frame were detected, whereas the method presented here processed, in the case of the prostate (Fig. 7), enables the handling of a larger number of detections as more than 700 events were detected in some frames. The tracking, used here, used a combination of the nearest neighbor approach and knowledge on the MB intensity, suggesting that it is not significantly inferior to the autocorrelation method. The tracking is in effect a sparse recovery method for the location of an MB path that has very few MB detections. In addition, there is no evidence in the literature that suggests that a high frame rate improves the statistics of the processing. Given that a bolus injection requires a minimum of a couple of minutes for the first pass to complete in most organs, it is suggested that it is this MB transit time, along with the dimensions of the vascular bed, that determine the MB number that is adequate to map the entire vascular structure. Here, it is suggested that mapping capillaries may be beyond the capability of image-based methods that aim at high resolution. Thus, vessels of tens of micrometers in diameter are the realistic targets of super-resolution ultrasound in clinical radiology, and most of these may be crossed several times in by the MB concentrations used here, thus providing adequate signal for processing. The inclusion of overlapping MBs is thus necessary. An approach that deals with the cumulative signal from all MBs in each pixel rather than particle events and, thus, uses the localization of spatially nonisolated MBs^77^ may be argued to include single and overlapping events in the processing. However, it is not evident from this processing what are the MB event areas, and thus it is much more difficult to assess detection and localization accuracies.

The super-resolution density maps presented here are the result of a robust detection and segmentation processes. All of the MBs were used in the tracking process including both single MBs of low intensity and overlapping MB events of large size and intensity. The signal enhancement through PPI and Haar-like features and the noise removal discriminating the background from the foreground make this algorithm capable of detecting even the weakest scatter. Christensen et al^24^ based the detection of single MBs on the cross correlation of each region in the reference frame and the subsequent frames. The groups that use ultrafast imaging detect individual events based on the correlation of each MB with the corresponding one in the next consecutive frame, due the high frame rate.^23,26,31,51^ These approaches are not effectively different to the one proposed here. Our approach enables the automatic detection and identification of MB events. Other methods apply the detection in B-mode frames after a similar approach to our background and foreground discrimination,^32,35^ thus dealing with multiple MB numbers. However, in CEUS, the shape of each scatterer in the image is nonregular and needs to be preserved. As a result, an optimal segmentation, using appropriately the watershed function, may be proposed for accurate localization of each event as well as ensure that nearly all MBs are detected.

Initial images of prostate cancer have been presented here from one patient. Although not conclusive, this initial result seems promising. Both density and velocity maps show good correlation with the histological evaluation. The velocity map suggests that tumor areas have redundant anastomotic vessels due to neovascularization. Extensive research aims at detecting and grading cancer by imaging technology to replace the use of invasive systematic biopsies (standard procedure) and reduce the risk of overtreatment and undertreatment. As aggressive (high grade) prostate cancer is correlated with angiogenesis and increased microvascular density,^78,79^ the proposed method may represent an asset for improved prostate cancer diagnostics and monitoring.

In future work, immunohistology by, for example, CD31 staining should be used to establish a ground truth of the microvascular architecture and improve over the adopted indirect comparison with the histology. A full study is required to assess this information that is otherwise difficult to compare with histological evaluation. The different microscopic techniques provided a criterion standard. However, these are limited, and this has become apparent through the improved near-microscopic resolution performance of ultrasound super-resolution images. Both microscopy histology and OPT images provided significantly reduced size measurements compared with the ultrasound density maps (Table 5; 41% mean difference), which confirms several other studies. It is known that optical imaging is undertaken ex vivo and after further tissue processing. This processing results in tissue shrinking.^80^ Further, fixation and histological preparation distort the tissues, and some of the variables required for this are difficult to standardize.^81,82^

The OCT performed in vivo provided the best comparison of vessel sizes with the density map with measurements within 10%. This confirmed that the measurements in the density maps are very accurate and demonstrated that ex vivo criterion standard comparison may be less appropriate for super-resolution ultrasound development. In addition, this OCT validation is superior to that using an in vitro setup with a capillary or capillary network that has been used previously.^23,30,32^ The in vitro setup provides lower PSF variability across the image compared with in vivo tissue imaging. This is because significant changes in the speed of sound across tissue affect the aberration and augment PSF variability compared with that of a translucent in vitro setup. This may be the reason that in vitro setups have been used previously to validate velocity estimation,^32^ as the diameter estimation is not a robust validation for the real imaging in vivo. In this context, the vessel diameter and thus system resolution are better validated in vivo. The 2 different-sized vessels (Fig. 5), which were embedded in tissue, had different vessel diameter and curvature, which provided a convincing validation of the vessel diameter accuracy of the ultrasound density maps at multiple positions in the same image. However, the most significant challenge with the use of the OCT was the matching of its plane with that of the ultrasound image. First, the ultrasound image is of low resolution and thus impossible to compare in situ. Second, the OCT is limited in depth (1.5 mm), which imposed an unusual position for the ultrasound transducer and restricted the imaging plane to very close to the surface of the ovary (and perpendicular to the plane in view for the rest of the imaging). However, it was shown that live techniques are more likely to be of use in the development of similar high-resolution ultrasound-based imaging methods.

In conclusion, a new super-resolution tool, which can be used with current clinical 2D CEUS, was presented. The feasibility was demonstrated in vivo for clinical radiology relevant image depths, with tissue motion and for short examination times under 2 minutes. The potential lies in the identification of regions with abnormal vasculature and particularly malignant tumors.
